# Low-Carbohydrate High-Fat Diet: A SWOC Analysis

**DOI:** 10.3390/metabo12111126

**Published:** 2022-11-17

**Authors:** Dena Nuwaylati, Basmah Eldakhakhny, Abdulhadi Bima, Hussein Sakr, Ayman Elsamanoudy

**Affiliations:** 1 Clinical Biochemistry Department, Faculty of Medicine, University of Jeddah, Jeddah 21959, Saudi Arabia; 2 Clinical Biochemistry Department, Faculty of Medicine, King Abdulaziz University, Jeddah 21465, Saudi Arabia; 3 Physiology Department, College of Medicine and Health Sciences, Sultan Qaboos University, Muscat 123, Oman; 4 Medical Physiology Department, Faculty of Medicine, Mansoura University, Mansoura 35516, Egypt; 5 Medical Biochemistry and Molecular Biology Department, Faculty of Medicine, Mansoura University, Mansoura 35516, Egypt

**Keywords:** low-carbohydrate diet, high-fat diet, ketogenic diet, obesity, weight loss, nutritional ketosis

## Abstract

Insulin resistance (IR) plays a role in the pathogenesis of many diseases, such as type 2 diabetes mellitus, cardiovascular disease, non-alcoholic fatty liver disease, obesity, and neurodegenerative diseases, including Alzheimer’s disease. The ketogenic diet (KD) is a low-carbohydrate/high-fat diet that arose in the 1920s as an effective treatment for seizure control. Since then, the KD has been studied as a therapeutic approach for various IR-related disorders with successful results. To date, the use of the KD is still debatable regarding its safety. Some studies have acknowledged its usefulness, while others do not recommend its long-term implementation. In this review, we applied a SWOC (Strengths, Weaknesses, Opportunities, and Challenges) analysis that revealed the positive, constructive strengths of the KD, its potential complications, different conditions that can make used for it, and the challenges faced by both physicians and subjects throughout a KD. This SWOC analysis showed that the KD works on the pathophysiological mechanism of IR-related disorders such as chronic inflammation, oxidative stress and mitochondrial stress. Furthermore, the implementation of the KD as a potential adjuvant therapy for many diseases, including cancer, neurodegenerative disorders, polycystic ovary syndrome, and pain management was proven. On the other hand, the short and long-term possible undesirable KD-related effects, including nutritional deficiencies, growth retardation and nephrolithiasis, should be considered and strictly monitored. Conclusively, this review provides a context for decision-makers, physicians, researchers, and the general population to focus on this dietary intervention in preventing and treating diseases. Moreover, it draws the attention of scientists and physicians towards the opportunities and challenges associated with the KD that requires attention before KD initiation.

## 1. Introduction

In 1960, Yalow and Berson defined insulin resistance (IR) as “a state in which a greater than normal amount of insulin is required to elicit a quantitatively normal response” [1]. Later, hyperinsulinism was addressed to explain the IR state. It was recognized as an endogenous pathophysiological mechanism, raised from IR and implicated in several metabolic and endocrine disturbances [2]. Today, it is documented that IR plays a critical role in impaired glucose homeostasis type 2 diabetes mellitus (T2DM), and the term metabolic syndrome (MetS) became evident [3].

MetS is a complex condition that constitutes central obesity, IR, hypertension, and dyslipidemia [4]. Due to the co-existence of these metabolic risk factors, MetS predisposes an individual to T2DM and cardiovascular disease (CVD) [5]. Normally, insulin promotes the uptake of glucose in the muscle and liver and inhibits lipolysis. While in IR, the insulin-mediated inhibition of lipolysis is impaired, which causes an increase in circulating free fatty acids (FFAs). This elevation of FFAs plays an essential role in the pathogenesis of MetS in various ways. FFAs inhibit the glucose uptake by muscles and enhance gluconeogenesis and lipogenesis, and it causes pancreatic β-cells toxicity, which impairs insulin secretion [6].

Alterations in any of the steps of the insulin signaling cascade can lead to IR, which causes dysregulation of intracellular signals that are usually dependent on insulin binding [7]. Activation of the insulin-dependent pathway promotes cellular glucose influx. However, this signaling cascade activation leads to the downregulation of gluconeogenesis and lipolysis simultaneously [8]. The mechanism of IR is not only related to an impaired insulin signaling pathway, it also includes the disruption of multiple metabolic pathways involving carbohydrates (CHO), amino acids, lipids, ketone bodies (KB), and even bile acids [9]. The metabolites of these metabolic pathways can affect insulin sensitivity directly and indirectly. The direct effect is mediated by modifying the mechanisms of the insulin signaling pathway, such as insulin receptor substrates (IRS). On the other hand, the indirect effect is mediated by changing the flux of substrates via lipogenesis, lipid oxidation, protein metabolism, and hepatic gluconeogenesis [3].

All of these metabolic changes contribute to the pathogenesis and development of several non-communicable diseases such as T2DM, CVD, non-alcoholic fatty liver disease (NAFLD), neurodegenerative diseases including Alzheimer’s disease (AD), and impaired lung function [10,11,12]. The prevalence of IR and MetS increases, with rates ranging from 20–40% in different populations, specifically in developing countries [13]. Many lifestyle and dietary factors contribute to IR and MetS, especially high insulin-stimulating diets (mainly simple CHO). Furthermore, there is an interplay between the genetic factors and the nutritional habits in the pathogenesis of IR and the individual components of MetS [14].

Moreover, obesity is one of IR’s significant consequences and comorbidities [15,16]. It is a severe health and economic burden and is one of the most common elements of preventable death [17]. Its treatment and prevention necessitate a long-term commitment to therapeutic strategies [17,18] and the adherence to a controlled regimen for weight maintenance, which is the most challenging part of a weight loss journey [18]. Caloric restriction is the first nutritional intervention in obesity treatment guidelines [19] and the management of IR state [20]. Hence, a dietary regimen low in CHO is preferable [21]. One of the low-carbohydrate diets (LCD) is the ketogenic diet (KD).

The KD is a very low-carbohydrate/high-fat and moderate-protein diet [22,23] that first emerged in the 1920s as a treatment for epilepsy in a regimen that resembles the concept of fasting [23]. Over the past 30 years, it has gained popularity for the management of obesity [23] and is considered a practical approach for rapid weight reduction [22]. Its application has also been evaluated in other medical conditions, such as T2DM, NAFLD, some types of cancers, AD, CVD, chronic kidney diseases (CKD), and pregnancy [24]. Years of researching the KD have brought on inconsistent viewpoints regarding its benefits, risks, and safety. Some researchers support its use due to its plentiful benefits [24,25,26] and others prove it is more harmful than valuable [24,27].

A SWOT (Strengths, Weaknesses, Opportunities, and Threats) Analysis Matrix has been successfully implemented in medical practice to evaluate various therapeutic regimens [28,29,30]. A qualitative study through a SWOT analysis was used to assess the KD [31]. To set “challenges” instead of “threats”, a SWOT analysis has been modified by some to be renamed a SWOC analysis with the same attributes: Strengths, Weaknesses, Opportunities, and Challenges.

To our knowledge, the majority of studies have addressed specific KD-related issues, or have generally highlighted its advantages and disadvantages, yet studies that fully address all aspects of a KD that aim to help in building a safe personalized strategy to implement it are lacking. This review illuminated the direct physiological effects of a low-carbohydrate/high-fat diet (KD) and subsequently performed a SWOC analysis; strengths and opportunities highlight the favorable features associated with the KD, while weaknesses and challenges show its potential drawbacks. The KD has undergone an age-old path of investigations. Nevertheless, concerns regarding its safety are still in question. We aim to answer some of the questions regarding this dietary regimen that has been an area of debate for years: Are ketogenic diets fruitful or hurtful?

## 2. Materials and Methods

The strengths, weaknesses, opportunities, and challenges (SWOC) associated with a KD are highlighted in this review. We searched for articles exploring the advantages and disadvantages of LCD with its various variations. Databases from which articles and reports were obtained were PubMed, PubMed Central, Cochrane Database of Systematic Reviews, MEDLINE, MedlinePlus, and Google Scholar database published from 2000 to 2022. The search terms included: low-carbohydrate diet, very-low-carbohydrate diet, high-fat diet, obesity, insulin resistance, insulin resistance-related comorbidities, metabolic syndrome, ketogenic diet, ketogenesis, advantages and disadvantages, physiology of ketogenic diet, and SWOT analysis. A total of 219 articles were cited: 97 review articles, 57 human studies, 48 animal studies, 8 official guidelines and books, and 9 meta-analysis and SWOT-based articles. The SWOT methodology highlights all the physical characteristics, associations, and interactions among the advantages and disadvantages of the KD. It also aims to reveal the positive, constructive strengths of KD that work together and the potential complications that need to be identified. It helps participants to decide on its use as a therapeutic regimen. Moreover, the SWOT analysis could highlight the opportunities of its use along with its associated challenges that physicians and participants should be aware of.

## 3. Physiology and Concept of the Ketogenic Diet

The KD is formulated to induce a state of physiological ketosis to energize the body with ketones rather than glucose [32,33,34]. Given that various ways can induce ketosis, several variations of the KD have been introduced, but with all of them adopting the same hypothesis of essentially restricting CHO intake. A standard very-low-carbohydrate ketogenic diet (VLCKD) mainly comprises CHOs that make up less than 50 g/day, around 75% fat, and a sufficient daily protein intake of 1–1.4 g/kg body weight [32,33,35].

The primary source of energy supply to body tissues is CHOs. Initially, when dietary CHOs are restricted, insulin secretion is significantly diminished, which reduces lipogenesis and fat storage [22,32]. At this point, gluconeogenesis takes over to provide the glucose needed for energy from non-CHO sources, mainly lactic acid and glycerol [22,32]. Unfortunately, gluconeogenesis-derived glucose fails to meet the body’s demand. As a result, and with the ongoing depleted insulin secretion and reduced fat storage, the primary source of energy is shifted to consuming fat through hepatic catabolism of fatty acids and KB produced by the liver [22,32,34]. Additionally, KB can pass the blood–brain barrier (BBB) to provide energy supplies to the central nervous system (CNS) [22,32,34].

**Figure 1:**
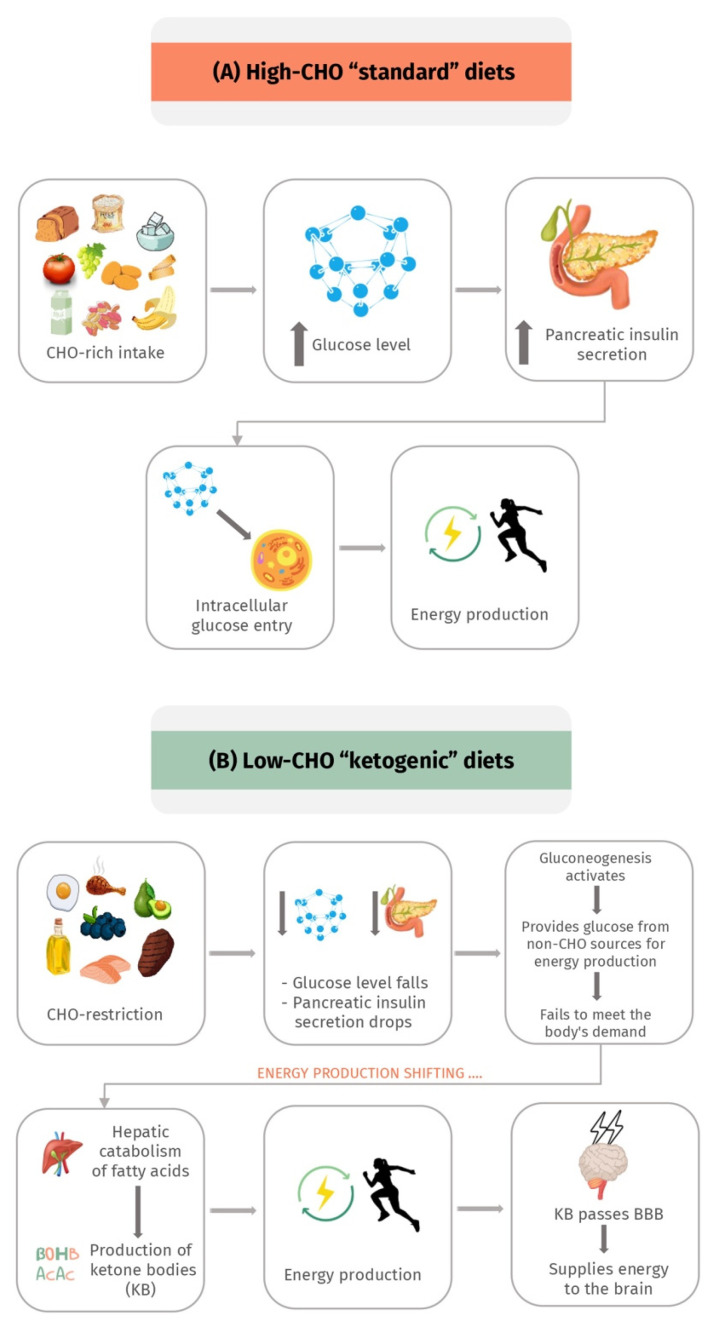
The physiological concept of the ketogenic diet; (**A**) represents the physiological effect of standard high-carbohydrates diets; (**B**) represents the physiological effect of low-carbohydrate ketogenic diets. CHO, carbohydrates; KB, ketone bodies; BBB, blood–brain barrier.

Unlike the life-threatening ketoacidosis associated with diabetes mellitus, this state is termed “nutritional ketosis”, which is benign, physiological, and safe due to the small amounts of KB produced (1–5 mM) that do not affect the normal blood pH of 7.4 [22,32,34]. In contrast, ketoacidosis is often associated with producing a hazardous level of KB, which shifts the blood pH [22,32,34]. The physiological effect of a low-carbohydrate KD in comparison to the high-carbohydrate standard diet is presented in Figure 1.

## 4. Strengths, Weaknesses, Opportunities, and Challenges (SWOC) Analysis of Ketogenic Diets

### 4.1. Strengths “S”

T2DM, ischemic heart diseases (IHD), dementia, and some types of cancers are examples of prevalent diseases that can be prevented by diet rather than drugs. The different mechanisms implicated in the pathogenesis of these chronic diseases include glycation, oxidative stress, mitochondrial dysfunction, IR, and inflammation [36,37,38]. This section explores the strengths of a KD and how it can alleviate these mechanisms.

#### 4.1.1. Ketogenic Diets Improve Insulin Sensitivity

The reduced response to insulin in the body is known as insulin resistance (IR) [39]. The primary etiology of hyperglycemia can predispose to increased glycation and oxidative stress [39]. The overconsumption of CHO and vegetable oils with the lack of physical activity that accompanies a modern lifestyle contribute to IR [39]. Insulin insensitivity triggers the pancreas to release more insulin for further blood glucose control [39]. The primary target organs for insulin action are the liver, adipose tissue, and skeletal muscles [39].

Insulin stimulates hepatocytes to decrease glucose production and increase de novo lipogenesis [39]. In the IR state, insulin fails to control the release of glucose in the blood as gluconeogenesis cannot be inhibited [39]. Hyperglycemia stimulates a compensatory release of insulin by pancreatic β-cells, which worsens IR [39]. At the same time, de novo lipogenesis continues to release triglycerides, which travels in the bloodstream in the form of VLDL-cholesterol or gets deposited within hepatocytes [39]. This metabolic disturbance leads to nonalcoholic steatohepatitis (NASH) [39]. Consequently, IR contributes to hypertriglyceridemia; and impairs the uptake of triglycerides (TAGs) into adipose tissue, increasing circulating TAGs [39].

Furthermore, insulin loses its ability to translocate Glut-4 onto the muscle cell surface, decreasing glucose uptake [39]. Decreased insulin level is associated with increasing its sensitivity [40]. Hence, the effect of dietary CHO restriction, including sugars and fructose-rich fruits, on IR has been extensively studied. KD has been shown to control blood glucose, decrease the need for insulin release [39], and improve IR by lowering the glycemic response generated by CHO in studies done on adult diabetic patients and in obese children and adolescents [41,42].

#### 4.1.2. Ketogenic Diets Decrease Glycation

Glycation is the process of adding a CHO to protein, lipid, or DNA [43]. It is an irreversible, spontaneous, and non-enzymatic process that can be considered normal and pathological. When it occurs between a CHO molecule and a free amino group in a protein, it forms glycated proteins called “Amadori” products [44]. These products can undergo further rearrangements leading to the production of advanced glycation end products (AGEs) [43,44]. It is not to be confused with glycosylation, an enzyme-controlled physiological process that occurs during the formation of glycoproteins [43]. Glycation can happen endogenously and exogenously [43].

Pancreatic β-cell dysfunction and the lack of blood glucose control cause a hyperglycemic state that enhances the glycation process, eventually leading to the loss or modification of protein function. AGEs accumulate in various tissues such as arteries, lenses of the eyes, and neurons [43], then promote atherosclerosis, cataract, dementia and aggravate various symptoms of diabetes [44,45].

KD stabilizes blood glucose levels and decreases its spikes, consequently improving AGEs. Restricting CHO intake in patients with IR and T2DM to <30% of daily calories improves the level of HbA1C, one of the glycation-derived AGEs [46]. Additionally, in another study, CHO restriction reduced AGEs in the kidney [47]. In addition, another study conducted on mice has looked at the long-term effects of CHO restriction and showed a significant reduction in different AGEs in the kidney [47].

#### 4.1.3. Ketogenic Diets Ameliorate Mitochondrial Dysfunction and Oxidative Stress

Mitochondria are required for metabolic balance in all multicellular eukaryotes because it is the central organelle responsible for cellular energy production [48]. Mitochondrial malfunction causes excessive weariness and other common symptoms in all chronic diseases [48]. This mitochondrial dysfunction can happen due to the loss of electrochemical transmembrane potential balance, malfunction of the electron transport chain (ETC), or the lack of metabolites transferring to mitochondria, which collectively can reduce adenosine-5′-triphosphate (ATP) production [49].

Oxidative stress is caused by a mismatch between cells’ creation and buildup of mitochondrial reactive oxygen species (ROS) and the biological system’s ability to detoxify these reactive products [50]. While high levels of mitochondrial ROS generation promote oxidative damage, low levels are essential for enhancing protective defense mechanisms, a phenomenon known as mitohormesis [36]. Protein phosphorylation, transcription factor activation, death, and immunity, are physiological processes that depend on a low amount of ROS formation and presence inside cells [51]. On the other hand, excessive ROS production negatively affects proteins, lipids, and nucleic acids [52]. The role of oxidative stress in various illnesses such as cancer, diabetes, metabolic disorders, atherosclerosis, and CVD, is well-known [52].

In 2014, a study on rats showed that KD enhances antioxidant defense, but insufficient evidence links the outcomes to mitohormesis [36]. At first, a KD can promote the production of small amounts of ROS, which subsequently activates the nuclear factor erythroid-derived 2 (NF-E2)-related factor 2 (Nrf2) antioxidant pathway, the main driver of detoxification genes [53,54,55]. The Nrf2 pathway was also activated by injection of KB [56].

The KD is thought to lower oxidative stress through a variety of methods. One possible mechanism is the scavenging properties of KB [57]. The circulating KB are Acetoacetate (AcAc) and β-hydroxybutyrate (ßOHB). They can scavenge various types of ROS, prevent ATP decline in neurons, and reduce ROS production by iodoacetate (IOA), a glycolysis inhibitor [57]. The addition of ßOHB and AcAc to glutamate-exposed dissociated neurons or calcium-exposed mitochondria, respectively, prevented O_2_ buildup and enhanced Complex I-driven state III respiration [58].

The KD also enhanced the production of the cytosolic and mitochondrial antioxidant proteins, superoxide dismutase (SOD-I & II), and NADPH Quinone Dehydrogenase 1 (NQO1) in a rat model [59]. These enzymes are O_2_ scavengers and under genomic control of Nrf2 signaling [60]. Moreover, the antioxidant glutathione (GSH) synthesis is elevated with KD consumption [53,54,55,61]. In the hippocampus of rats, ßOHB was observed to prevent hypoglycemia-induced lipoperoxidation, which is an index of oxidative damage [57]. Additionally, KD raises the total antioxidant capacity, glutathione peroxidase (GPx), and catalase activities were raised in the hippocampus homogenate of rats [62]. Moreover, animals with higher blood ßOHB concentrations (1.2 mM) were protected from paraquat-induced oxidative damage to proteins and lipids, as seen by a decrease in protein carbonyls, the toxic compound 4-Hydroxynonenal (4-HNE), and lipid peroxides levels [63]. Therefore, KD can enhance mitochondrial functions, activate various protective antioxidant pathways, and decrease oxidative stress by eventually reducing the production of ROS [54,55,61].

#### 4.1.4. Ketogenic Diets Have Anti-Inflammatory Effects

Inflammation is accompanied by activating various immunological and non-immunological cells that protect host cells from pathogens [38]. The effect of a KD in ameliorating inflammation was proven in multiple human and animal studies. The KB mediate their anti-inflammatory effect by inducing the release of anti-inflammatory cytokines and lowering pro-inflammatory ones [64,65,66]. ßOHB was shown to suppress the stress-related inflammasome production in the endoplasmic reticulum [67,68]. Moreover, ßOHB transport to the brain via the BBB is facilitated by the unique expression of its transporter, monocarboxylate (MCT1), on endothelial cells [69]. Once in the brain, ßOHB not only supplies energy but also activates the hydroxy-carboxylic acid receptor 2 (HCA2), reducing neuroinflammation [69]. KD has also been found to induce HCA2 and create a neuroprotective phenotype in bone marrow-derived macrophages that infiltrate the brain [70]. HCA2 suppresses the inflammatory promoter nuclear factor kappa-light-chain-enhancer of activated B cells (NF-κB) in macrophages [71]. Moreover, it activates the neuroprotective macrophage phenotype similar to Prostaglandin D2 (PGD2) synthesis by Cyclooxygenase 1 (COX1) [72] which provides anti-inflammatory and neuroprotective effects [73]. Additionally, PGD2 metabolite inhibits IkappaB kinase (IκB kinase or IKK), the primary activator of NF-κB [71]. Furthermore, the fasting-like state induced by KD contributes to neuroprotection by increasing corticosterone levels, enhancing apoptosis of autoreactive lymphocytes, exaggerating autophagy, and oligodendrocyte regeneration [74].

#### 4.1.5. Ketogenic Diets Combat Malignancy-Associated Features

Primarily, cancer cells feed on glucose to survive and proliferate through accelerated glycolysis, a phenomenon known as the “Warburg effect” [75]. The KD has been studied as a simple, tolerable, and non-costly way to combat this effect and delay tumorigenesis [53]. Studies have also revealed that the KD can protect healthy cells from the toxic effects of chemotherapy while promoting toxicity in cancer cells [76]. In addition, the KD can diminish a tumor’s growth and induce an anti-inflammatory effect in mice [53,77]. Studies have also proven that ketones and a KD can have an anti-brain cancer effect by promoting a metabolic state with anti-inflammatory, anti-angiogenic, and pro-apoptotic properties that helps to shrink tumor growth in the brain [78,79]. The insulin-activated phosphoinositide-3-kinase (PI3K) pathway regulates the proliferation and differentiation of cells and controls various metabolic pathways [80]. Mutations in the PIK3CA gene, encoding PI3K, lead to its activated signaling and are considered a hallmark of cancer [81,82]. Selective PI3K inhibitors have been studied as a promising anti-cancer drug [83]. The pharmacological inhibition of PI3K disturbs glucose homeostasis and leads to a state of transient hyperglycemia that can be rapidly resolved by the pancreatic insulin feedback [84,85]. However, in patients with IR, hyperglycemia may be exaggerated that requires treatment cessation [84]. It has been believed that the PI3K inhibitor-induced insulin feedback weakened the treatment effect by reactivating PI3K signaling [84]. Studies have shown that cancer patients who are candidates for PI3K inhibitors might benefit from the KD as it interrupts hyperinsulinemia, which might boost the effects of PI3K inhibitors [53,84].

#### 4.1.6. Ketogenic Diets Improve Blood Lipid Parameters

Various guidelines have proposed that the ideal diet for cardiovascular (CV) risk reduction is a low-fat diet (7–10%); hence, prescribing low-fat diets with nearly 60% of calories obtained from CHO is a standard for CV patients [86,87]. Consuming fat-rich diets has been believed to adversely affect cardiovascular outcomes, and saturated fats mainly increase low-density-lipoprotein cholesterol LDL-C, thus promoting intravascular fat deposition [86]. LDL-C and total cholesterol (TC) were previously considered the best biomarkers to assess CV outcomes, but nowadays apolipoprotein-B (apo-B), TC/HDL ratio, small dense LDL particles (sdLDL) have come out as biomarkers with better value in assessment [88]. Accordingly, researchers investigated the effect of macro-nutrients on other CV biomarkers [89].

A study in 2006 on 178 men that analyzed the effect of the dietary intake of around 25% CHO with a 7–10% of saturated fats, found that the increase in LDL-C was secondary to elevated large-sized LDL particles, which are less atherogenic [90]. In contrast, the more atherogenic sdLDL was lowered [90]. Moreover, another prospective-cohort PURE study has proven that higher saturated fat does have an LDL-C elevating effect. Yet, it lowered triglycerides (TG) and TC/HDL ratio, increased the protective HDL levels, and a CHO-restricted diet with high fats had effects on atherogenic biomarkers that are contradictory to other conventional beliefs [91]. For a good view of the effects of KD on CV biomarkers, studies have explored CV outcomes with nutritional ketosis induced by a daily CHO intake of <50 g. They found that KD remarkably reduced TG with no significant alterations in TC, LDL, or HDL [92,93]. Additionally, an LCD was seen to ameliorate dyslipidemia associated with MetS, as observed biochemically and histologically in rats [94].

Along these lines, the findings mentioned above have highlighted that the conventional lipid profiles might not be the best indicator of KD effects on CV outcomes, and the analysis of comprehensive lipid fractions might be necessary for proper assessment.

#### 4.1.7. Effects of Ketogenic Diets on the Epigenome

Epigenetics is the study of how non-genetic factors alter or modify gene expression. Alterations such as DNA methylation and histones modification can be environment driven [95]. Studies have shown that diet causes epigenetic modifications later in life [96,97,98]. Certain types of low-carbohydrate (ketogenic) foods, such as fibers, berries, and rich in long-chain fatty acids, were shown to positively impact epigenetic alterations like methylation patterns [95] by increasing adenosine levels, which blocks DNA methylation [99,100]. ßOHB upregulated detoxifying genes by inhibiting class I and II histone deacetylases (HDACs) in a dose-dependent manner, resulting in increased histone acetylation regardless of whether ßOHB is raised by fasting, calorie restriction, or infusion [61,63]. These findings have driven the interest of researchers to explore KD’s effects on other epigenetic activities, such as telomeres’ length and chromatin structure [86].

#### 4.1.8. Ketogenic Diets and Gut Microbiota

A KD modifies the gut microbiome’s composition to prevent inflammation and diminish IR. It is also reported that the KD decreased Firmicutes and increased Bacteroidetes in a study done on a murine model [101]. Moreover, it increased *A. muciniphila*, *Parabacteroides* spp., while decreasing alpha diversity in another study on mice [102]. Another study showed that it increased *Akkermansia muciniphila* and *Lactobacillus* and decreased the inflammatory bacteria *Desulfovibrio* and *Turicibacter* [103]. In addition, the KD-induced ßOHB, which has anti-inflammatory effects, is due to its ability to inhibit the NLRP3 inflammasome [77]. Moreover, ßOHB modifies microbial-mediated immunomodulation and directly influences immune responses [104].

The impact of the KD on inflammatory bowel disease (IBD) has been also looked into, yet the results were inconsistent between studies. In colitis-induced mice models that were fed with either a KD, LCD, or a normal diet, the KD was shown to distinctly alleviate inflammatory colitis by altering the gut microbiota, increasing *Akkermansia*, protecting the intestinal barrier function, and decreasing the formation of various inflammatory cytokines and RORγt + CD3^–^ group 3 innate lymphoid cells (ILC3s) [105]. These findings might have reflected an opportunity for the KD as an adjuvant therapy for treatment of IBD; however, other studies have shown contradictory effects. In another mouse model with induced-colitis, the KD significantly aggravated the disease, induced weight loss, shortened colon length on histological examination, upregulated various inflammatory cytokines, increased GUT permeability, and reduced the expression of genes involved in intestinal epithelial barrier which disrupted its protective functions [106]. The impact of the KD on IBD has not been extensively studied though and might be an interesting area for future research [107].

### 4.2. Weaknesses “W”

Although the KD reduces body weight and improves IR, it has poor long-term tolerability from its various side effects. Adverse effects can be short-term and described as “keto flu” by the public [108]. Keto flu constitutes a temporary phase of symptoms experienced by around 30% of individuals within the first few weeks that follow the initiation of the KD [108]. It is accompanied by gastritis, diarrhea or constipation, gastrointestinal pain, nausea, bloating, headache, muscular cramps and weakness, dizziness, body aches, difficulty concentrating, and fatigue [108]. Why keto flu is experienced by some and not all individuals is unknown; however, the electrolytes imbalance with high sodium, potassium, and water losses associated with CHO withdrawal are believed to be the keto-flu promoters [109]. Furthermore, with KD initiation, people with diabetes may experience hypoglycemic attacks if they are non-compliant with their medications [110]. Some studies conducted in geriatric- and pediatric-age groups have shown that after the first month of following the KD, long-term adverse effects have also been reported. These adverse events include cardiomyopathy, osteopenia, iron deficiency anemia, nephrolithiasis, hepatic steatosis, renal stones formation, and nutritional deficiencies [111,112,113]. The rare KD-associated cardiomyopathy could be explained by the prolonged poor dietary intake and food refusal that causes energy failure that leads to deficiency of minor elements, such as selenium [114,115], and electrolytes disturbances, such as hypokalemia [108], which are both responsible for cardiomyopathy [108,114,115]. Moreover, the loss of bone mineral density induced by prolonged KD intake have been proposed to be caused by bone microstructural abnormalities that promote bone absorption via activation of osteoclasts, rather than inhibition of bone formation mediated by osteoblasts [116,117]. Additionally, the KD negatively affected athletes’ bone remodeling and modeling markers [118]. This section throws a beam of light on further details of the main weaknesses and harms of a KD.

#### 4.2.1. Poor Weight Maintenance and Rebound Weight Gain

Hyperinsulinemia is believed to promote weight gain in various ways. Hyperinsulinemia stimulates the uptake and storage of glucose within the adipose tissue, liver, and muscles, ending in weight gain [119]. It also elicits a sodium retention effect by activating the renin–angiotensin–aldosterone system, which enhances sodium absorption from renal tubules, subsequently expanding extracellular fluid volume and causing weight gain [119,120]. Since food intake triggers glucose metabolism and simultaneous insulin release, the effects of LCD on insulin levels have been explored [121]. CHO restriction was accompanied by a lower pancreatic insulin release [119,121], inhibiting renal tubular sodium and water retention [119]. The majority of people following a KD notice an initial rapid weight loss of 4–5 kg within the first two weeks, mainly from alleviating the action of insulin on renal tubules, causing fluid excretion [22,120]. However, at least 18 weeks of adherence to a KD is required to obtain the target weight by losing fat and excess retained water [121]. Various reasons to discontinue a KD have been documented, such as the reluctance to cut out CHO, the poor control of children’s dietary habits, medical reasons interfering with compliance, and issues related to caregivers providing the diet [122]. Moreover, the use of dietary interventions in general for weight loss management is subject to rebound weight gain after their initial success in weight reduction; hence, achieving long-term weight maintenance might be difficult for the majority of obese individuals [123].

#### 4.2.2. Nutritional Deficiencies

CHO could be considered an essential source of various vitamins and minerals [124]. Losing weight requires macronutrients restriction for a particular duration, which can sometimes be long enough to cause nutritional deficiencies [125]. Thiamine (vitamin B1), folate (vitamin B9), magnesium, calcium, and iron, have all been reported to be deficient following KD initiation [125]. Excluding dairy products from some KD variations leads to iodine deficiency [125]. Life-threatening conditions have been reported with long-term KD consumption, such as optic neuropathies [112] and Wernicke’s encephalopathy [126]. Additionally, maternal folate deficiency from a KD may increase the risk of fetal deformities [127,128]. Moreover, KD-induced osteopenia can be explained by the KD-associated vitamin D deficiency related to malnutrition from diet intolerance that eventually contributes to bone demineralization [114,116,117].

#### 4.2.3. Electrolyte Disturbances

Once CHO is dramatically reduced from the diet, electrolytes are managed differently by the body. According to the Centers for Disease Control and Prevention (CDC), sodium and potassium are critical minerals needed by the body to function appropriately, and they work simultaneously. When KB are utilized as a source of energy instead of glucose, insulin level drops [108]. Hypoinsulinemia is associated with the inability to retain salt and water by the kidneys, according to the Cleveland clinic, which leads to the loss of sodium in the urine, and the subsequent hyponatremia [40,108,111]. Moreover, due to the close interconnection between sodium and potassium, urinary potassium excretion increases, leading to hypokalemia [40,108,111,129]. This effect is usually accompanied by other electrolyte disturbances, which collectively contribute to “keto flu” symptoms explained earlier [108,111].

#### 4.2.4. Renal Stones and Renal Impairment

Researchers have warned that while low-carbohydrate/high-protein diets promote short-term weight loss, this type of diet could be less effective in the long run and may even be harmful to overall health. Protein-rich foods can also be rich in fats, which raises the risk of heart disease and T2DM. LCD, such as those rich in fruits and vegetables, are linked to a high predisposition to renal stones formation [130]. Studies on healthy individuals have observed that a six-week LC high-protein diet elevated the acid load on the kidneys, predisposing to nephrolithiasis. Animal protein has been proven to increase oxalate excretion in the urine, a molecule that forms kidney stones when combined with calcium and other substances [131]. Children who followed the KD for a prolonged period to suppress persistent grand mal seizures are predisposed to a higher risk of calcium oxalate and uric acid renal stones [132]. This danger in an adult undergoing a protracted ketogenic attempt is similarly substantial [130]. On the other hand, other studies have shown that the risk of KD-associated nephrolithiasis can be diminished if the KD is implemented in a perfect and standardized protocol with strict and continuous follow-up [110,133]. A KD formulated with a moderate daily protein intake of 0.8–1 g/Kg (90–150 g/day) as indicated in the Atkins diet closely resembles what an average adult usually consumes [110] and is generally well-tolerated with no adverse effects on bone, kidneys, or other health indicators. Protein intakes of more than the maximum suggestion of 1 g/Kg were assumed to have a harmful influence on health. While some evidence exists that more significant protein consumption can raise glomerular filtration rate [133], there is no evidence that this normal physiologic response is linked to gradual kidney function loss in healthy individuals [134].

#### 4.2.5. Effects on Embryonic Growth during Pregnancy

The KD has been used by childbearing-aged women during pregnancy for weight control; however, its safety as a gestational diet have been questioned. A poorly formulated gestational diet might negatively impact fetal developmental adaptation, predisposing to various disorders later in life [135]. Thus, the KD was studied in a murine model for its effects on prenatal embryonic organ development [135,136]. The development of multiple organs in mice embryos whose mothers were fed the KD was compared to the embryonic development of those on a standard diet [135]. Significant alterations in embryonic growth were observed in those on the KD as seen by measuring the embryonic and organs volumes’ percentages [135]. By the end of organ development, the larger percentage of the embryos was occupied by the brain, heart, and liver, which showed subsequent volume reduction by the time of parturition, and a further reduction of postnatal organs volume as compared to an average adult mouse [135]. These changes can be explained by different energy substrate preferences in various organs and can predispose to later abnormal functions [135]. The KD was also shown to reduce maternal fertility and increase the risk for fatal ketoacidosis in another mouse model [136]. The American Diabetes Association (ADA) recommends a non-ketogenic high-energy diet for women with gestational diabetes [137]. This is because the formation of KB normally increases during the third trimester due to pregnancy-associated IR and the higher fetal demand for energy [138]. Ketones are able to passively diffuse from the mother to the fetus by crossing the placenta, and maternal KB elevation was shown to increase the risk of fetal cardiac abnormalities, reduced intelligence, and oligohydramnios [138]. Despite the rare documentation of the KD effects on humans during pregnancy and breastfeeding, life-threatening ketoacidosis was reported on a lactating mother on a strict ketogenic diet [139].

#### 4.2.6. Growth Retardation in Children

Another adverse effect of the KD is short stature in children [140]. The growth of these children was observed to fall into the tenth percentile or lower, which implies that something is fundamentally wrong with sustained ketosis that impairs average growth [141]. Other studies have found no adverse effects on children’s growth and development who followed a short-term KD for less than six months [142,143]. In spite of that, other studies on the long-term implementation of KD have reported either a decrease of height alone [144] or of both weight and height [145]. This potential growth retardation effect seen with long-term KD consumption can be explained mainly by inadequate caloric intake [146]. Such inconsistencies indicate that this concern deserves further analysis [140]. Furthermore, insulin is essential for preserving the response of liver to growth hormone (GH), and the reduction of hepatic GH receptor (GHR) expression is associated with insulin deficiency [147], and the KD was observed to suppress the expression of hepatic GHR in rats and impair the central regulation of GH release [148], which could be another explanation to the retarded growth in children fed with the KD.

#### 4.2.7. Effects on Intestinal Flora

Despite the previously highlighted benefit of the KD on the gut microbiome, certain KD variations may have opposing effects. Lifestyle aspects such as sleeping patterns, exercise, antibiotic usage, and even nutrition may influence the genetic make-up of a microbiome. Because these bacteria differ in their capacity to collect energy from food, they can modify our reaction to diverse food sources, influencing the postprandial glucose response (PPGR) [86]. It is vital to figure out how the gut microbiota and nutrition interact, how that connection relates to general health, and whether new dietary changes, like the KD, would have a good or a detrimental impact overall on the microbiome needs to be explored. Whole grains are essential for overall health and play a vital role in forming a healthy microbiota [149]. As a result, LCDs are more likely to be nutritionally deficient due to the lack of fibers, essential vitamins, minerals, and iron [150]. Moreover, they might be accompanied by insufficient intake of whole grains needed to maintain a healthy microbiota [150]. This hypothesis is based on The Continuing Survey of Food Intake by Individuals (CSFII) that looked at nutrient intake while consuming various quantities of CHO [151]. Strong evidence regarding the long-term consequences of the KD on gut flora is currently limited.

#### 4.2.8. Ketone-Proteins Adducts Formation

As explained in a previous section, one justification for using the KD is minimizing glycation adducts that promote inflammation. However, an overlooked risk associated with the KD is related to the reactivity of KB toward proteins [152]. While excessive glucose reacts with proteins, causing AGE products that promote inflammation and vascular damage, ketones were found to generate the same modifications in proteins leading to the same glucose-associated vascular damage and by the exact same mechanisms [152], which may have adverse health outcomes.

### 4.3. Opportunities “O”

Despite the escalating evidence of the various drawbacks of the KD, its proven positive role in multiple disorders made KD an attractive approach to be further explored in several areas. The most critical conditions in which KD has provided an opportunity to positively contribute to their outcomes will be outlined in this section.

#### 4.3.1. Weight Loss

For any dietary regimen to be successful, strict adherence is essential. When LCD was compared to a low-fat diet and a Mediterranean caloric-restricted diet over two years, the highest weight loss was observed among those following an LCD, especially when their diet was initiated by a complete two months of nutritional ketosis (<20 g/day) with increasing CHO over stages [153]. While various diets rely on caloric restriction, a KD provides an ideal opportunity for those who are unable to restrict their calories since it has the benefit of promoting weight loss while eating with satisfaction [86]. Despite the non-caloric-restriction nature of a KD, it provides the weight loss benefits of an LCD [86].

#### 4.3.2. Personalization with Safety

Like any other therapeutic regimen, a KD may be suitable for some individuals more than others and contraindicated in several situations. The expanding body of research conducted over the past years has highlighted the physiological and molecular mechanisms by which KD functions; therefore, it has given the opportunity to safely implement a KD that is personalized for each individual at the lowest possible risk [154]. Additionally, the availability of well-trained health care practitioners with appropriate awareness of the KD-associated safety alerts, and updated knowledge of the recommendations necessary to initiate a safe and effective regimen, has also lowered the chances of inappropriately following an unsuitable weight loss regimen that has more losses than gains [154].

The many variations of the KD enabled more effective methods to be implemented based on patients’ age, behavior, preferable amounts of certain macronutrients, and their desire to follow an on and off dietary course [155]. For example, the medium-chain triglyceride ketogenic diet (MCTD) allowed children to follow the KD due to its higher CHO and protein content with a lower fat intake. Targeted ketogenic diets (TKD) is another variation that allows the consumption of CHO around the workout time for those performing physical exercise to maintain a better performance without altering their ketotic ketonic state [156]. At the same time, cyclical ketogenic diets (CKD) permit periods of high CHO consumption to alternate with periods of a classical KD [156]. The intervening CHO-rich periods replenish muscle glycogen stores to sustain the function of exercising muscles [156,157].

#### 4.3.3. Glycemic Control and Diabetes

Given the well-explained role of the KD in improving insulin sensitivity, it provided an excellent opportunity for glycemic control and the treatment of IR-associated conditions. By lowering the glycemic response generated by CHO and improving IR, the KD can improve both insulin-dependent diabetes mellitus (IDDM/T1DM) and T2DM [27,41,42,87]. When the effects of CHO-restricted KD were assessed in various studies, and among those with T2DM and non-diabetics with different degrees of obesity, the KD was able to lower fasting plasma glucose, improve insulin sensitivity, and improve glycemic control as observed by hemoglobin A1c levels [158,159,160]. Hence, the KD has provided an opportunity to reduce the need for anti-diabetic medications [158,159,160].

Currently, there is limited evidence in the literature regarding the effects of the KD on T1DM, yet studies have proven that those with T1DM could also benefit from the KD. In a randomized crossover study that compared LCD to high-carbohydrate diet over 1 week in T1DM participants on insulin pump therapy, those on LCD had lower mean blood glucose levels [161]. LCD had significantly lower average daily blood glucose levels, euglycemia, less glycemic variability, and a lower need for daily doses of insulin [161]. Other studies on T1DM patients following the KD have also reported good glucose control with a nearly normal HbA1C level (5.3–5.7%), a low rate of severe adverse events, and a higher satisfaction [27,87]. However, many patients have also reported frequent hypoglycemic episodes [27], which indicates that close blood glucose monitoring in those patients is essential.

#### 4.3.4. Adjuvant Therapy in Cancer

As clarified in an earlier section, KD combats the Warburg effect, delays tumor growth, and elicits anti-inflammatory, anti-angiogenic, and pro-apoptotic features associated with malignancies [53,78,79]. Various studies have explored the effects of a KD on different types of malignancies and proved its role as a novel therapeutic approach in such cases. Gastric cancer is one of the cancers that have been shown to benefit from a KD are gastric cancers; when mice were injected with human gastric adenocarcinoma cells and subsequently fed with a KD, tumor growth was delayed as compared to mice consuming a standard diet [162].

Glioblastoma, an aggressive type of brain cancer, has been associated with a short survival duration after the failure of conventional anti-cancer therapies [163,164]. Glioblastoma cells are highly dependent on glucose for obtaining the energy needed to proliferate. Hence, LCDs have lengthened glioblastoma patients’ survival time [164,165]. Studies have shown that Glioblastoma cells cannot consume ketones as a source of energy when glucose is absent due to their low expression of ketolytic enzymes, unlike normal brain cells [163,164]. Therefore, nutritional ketosis selectively arrested cancer growth and did not elicit the same function on normal brain cells [164,166].

The KD has been studied on various cancer models, such as pancreatic, breast, endometrial cancers, and leukemia, as an approach to prevent the unwanted insulin feedback in patients treated with PI3K inhibitors, which indicated that the KD helps to overcome the drug resistance [53,84]. These findings suggested the opportunity a KD provides for cancer patients in augmenting the efficacy of their treatment [53,84].

#### 4.3.5. Neuroprotection

It is known that excessive ROS and mitochondrial dysfunction are central features of degenerative brain diseases, which result in harmful cerebral effects, including DNA, lipid, and protein damage. The KD is believed to provide a great opportunity for neuroprotection through its anti-inflammatory and antioxidant effects, in addition to its ability to ameliorate mitochondrial dysfunction as previously explained. These effects are primarily due to nutritional ketosis, reduced blood glucose concentration, and decreased insulin/glucagon ratio [113]. Another possible mechanism of KD-associated neuroprotection is its energetic property. ßOHB has been shown to provide more energy for the brain per oxygen unit than glucose [167]. The induction of antioxidant enzymes in the hippocampus of experimental animals by KD-induced nutritional ketosis has also been reported.

Nutritional ketosis has provided an option to control seizures as a non-pharmacological treatment in various forms of medication-refractory epilepsy [32,168]. The multiple mechanisms by which the KD acts to improve brain functions have enlightened the prospect of using the KD in other neurological disorders [113]. The KD has been investigated in animal and clinical studies to treat some neurological and neurodegenerative diseases and provided good results. These neurological diseases include amyotrophic lateral sclerosis [169], traumatic brain injury (TBI) [170], and neurodegenerative diseases such as Parkinson’s and AD diseases [79,171].

One of the most promising opportunities for treating AD is the KD [32]. It was observed that a long-term administration of KB has a protective and therapeutic role in treating AD patients [172]. The KD was shown to decrease the production of Amyloid Precursor Protein (APP), and consequently the amyloid peptide, as well as activate the peroxisome proliferator-activated receptor gamma (PPAR), which ultimately suppresses systemic inflammation [173]. Moreover, the KD improved the cognitive functions of an experimental mouse model of AD by reducing beta-amyloid and highly phosphorylated tau proteins in its brain. The benefits of the KD were also proven concerning the motor functions of the experimental animals [174,175]. Enhanced apoptosis associated with mitochondrial dysfunction is the cornerstone of AD pathogenesis [176], and the KD was seen to inhibit apoptosis in the hippocampus and stabilize nerve-cell synapse functions [177].

It was shown that ßOHB acts in vitro as a neuroprotective agent against 1-methyl-4-phenyl-1,2,3,6-tetrahydropyridine (MPTP) toxicity on dopaminergic neurons [174]. MPTP is responsible for the death of dopaminergic substantia nigra cells, producing a syndrome identical to Parkinson’s disease. In addition, beta-hydroxy-lactate reduced the neurotoxicity of MPTP by improving cellular respiration, ATP production, and consequently enhanced motor skills, together with an increased dopamine volume in the mesencephalon [178]. Hence, Parkinson’s disease patients have also gained benefits from nutritional ketosis.

Moreover, the KD and KB can aid the regeneration of demyelinated axons, the primary pathology of multiple sclerosis (MS) [179]. Hence, it has shown promising results in managing MS, especially the relapsing/remitting type [180,181]. The inhibitory effect of the KD on low-grade inflammation and neuroinflammation and its immunomodulatory effects explain its role in treating MS [182].

#### 4.3.6. Cardiovascular Risk Factor Control

CHO restriction was shown to control various cardiovascular risk factors; hence, it can be an excellent chance for those with higher risks for CVD. The known positive effects of the KD on blood lipids were observed even with high saturated fats intake [183]. As various studies have established, the KD can lower TAGs and total cholesterol while elevating the atheroprotective high-density lipoprotein cholesterol (HDL-C) [184,185]. The previously highlighted shifting of LDL size with the KD to the larger, less atherogenic LDL was the most interesting [183]. However, controversy persists; studies have demonstrated that a low-carbohydrate, high-fat diet may worsen the lipid profile in diabetic patients, although glycemic control improved with hypoglycemic medications [27]. Moreover, a meta-analysis of randomized controlled trials has analyzed the effects of the KD on CV risk factors, including blood pressure [186]. A more considerable reduction in diastolic blood pressure (DBP) was observed in groups following a KD than those on low-fat diets [186]. In contrast, another meta-analysis found no differences in systolic or diastolic blood pressures between those on a KD and those on a low-fat diet [187]. Whether CHO restriction controls blood pressure cannot be ascertained. However, whether these positive effects of the KD on CV health are sustainable in the long run without causing harm is unknown.

#### 4.3.7. Treatment of Polycystic Ovarian Syndrome

Polycystic ovarian syndrome (PCOS) is a common endocrinological disorder of reproductive-aged women [188]. It is presented with menstrual irregularities, hirsutism, infertility, and excess androgens [188]. PCOS is linked to obesity, and other metabolic disturbances, such as IR/T2DM, dyslipidemia, hepatic steatosis, and MetS [188]. Its pathophysiological mechanism is mainly based on excess secretion of androgens from ovaries and adrenal glands that is due to impaired ovarian steroid hormones’ synthesis as well as hyperinsulinemia [189]. IR is a key feature in those with PCOS [189]. In tissues where steroidogenesis occurs, in this case ovaries and adrenal cortex, insulin is necessary to augment the effects of hormones that promote steroid hormones’ synthesis. Therefore, the IR and hyperinsulinemia associated with PCOS trigger excessive steroidogenesis, causing increased testosterone levels [189]. These mechanisms are confirmed by the observed clinical improvement in patients with PCOS with weight reduction and therapeutic interventions that sensitize insulin [189]. Hence, weight loss and improving IR are part of the therapeutic interventions for PCOS treatment.

Generally, successful weight management in women with PCOS face some obstacles as they are more prone to resistant weight gain than others, and their excess androgens enhance abdominal adiposity [189,190]. Women with excess androgens have also reported increased craving to CHO, which increases their dietary intake [191]. Accordingly, several diets have been tested for their efficacy in weight reduction among those women, and some were found to be more ideal than others [192,193,194].

The KD has been shown to have positive effects on women with PCOS. A study has implemented a strict KD on women of reproductive age with PCOS, BMI >27 kg/m^2^, and no other severe medical disorders for the treatment of PCOS [192]. Their fasting serum insulin and testosterone levels have significantly dropped after a six-month follow-up, as well as a remarkable weight reduction [192]. Another randomized study comparing the KD with a standard diet in women with PCOS has found that the KD improved IR, and reduced blood glucose, fasting insulin, and testosterone levels [194], positively impacting their medical condition. However, these findings were limited by their small sample sizes; therefore, they cannot be relied on for general applicability.

#### 4.3.8. Alleviation of Inflammatory Pain

Physiologically, acute inflammation protects the body from various pathogens and enhances tissue repair [195]. While chronic inflammation serves no protective mechanisms and may elicit tissue damage that might be associated with pain [195]. In this situation, the activation of various proinflammatory mediators causes the sensitization of neurons associated with peripheral pain [195], leading to the so-called “inflammatory pain”. According to the known interrelation between diet and inflammatory pain [196,197], recent studies have looked over the chances of KD as a therapeutic strategy for inflammatory pain [198]. When inflammation was induced in mice and rats’ models treated with a KD, tactile allodynia was vastly diminished [198]. This finding supported another study on allodynia associated with MetS, which improved with a KD and enhanced peripheral neural regeneration [199]. Neuropathic inflammatory pain can be central or peripheral, and both can benefit from the KD, yet the response may vary between conditions [198]. The use of the KD as a therapeutic strategy for the pain of various causes was successfully reported in several other conditions, such as migraines [200] and IBD [201]. However, the exact mechanism by which a KD improves pain is still indefinite. Finally, various clinical and pre-clinical studies have highlighted that a KD’s positive role extends beyond weight loss. The KD can offer more comprehensive therapeutic options, and more studies are encouraged to explore its concealed benefits.

### 4.4. Challenges “C”

The strengths, weaknesses, and opportunities of extreme CHO restrictions are now well-appreciated. However, and like several therapeutic approaches, the KD has faced many challenges, and the most important will be discussed in this section. The quality of diet could be primarily affected by a KD. Eliminating CHO is not confined to bread, rice, and pasta [24]. Essential fibers, iron, vitamins (A, B1, B6, B9, E and K), and minerals (potassium, magnesium and calcium) may all be insufficiently provided in the diet as CHO-containing fruits, vegetables and legumes should be taken into consideration [24,202,203]. Therefore, micronutrients inadequacy is widespread [204], and keeping up with long-term supplements to overcome some of these shortfalls is challenging.

Another significant factor affecting the obtainment of favorable results and proper assessment of the outcomes is the compliance to the KD, which can partly be secondary to the limited food choices that make the KD intolerable in the long run [110,205,206]. Other than food intake records, serum ßOHB and urine KB levels are common ways to monitor the response to the KD and assess diet adherence [207,208]. Serum ßOHB and urine KB levels may be inaccurate when the KD is not maintainable and are obtained at additional costs [110,206].

Since some patients follow the KD as a therapeutic intervention for a particular disease, not just for weight loss, restricting certain foods that patients find delightful might be more stressful for both patients and their caregivers. Hence, adjusting the suitable KD option without interfering with diet compliance is challenging [163]. Furthermore, enjoying socializing with people while on a KD without messing up the planned diet is another dilemma [163].

It has been reported that in some hospitalized patients, or those receiving medications that induce hyperglycemia, hidden CHO might hamper the effect of nutritional ketosis [168]. For instance, drug-induced hyperglycemia may be caused by β-blockers, thiazide diuretics, glucocorticoids, and some antipsychotics [209,210]. Moreover, lorazepam, for example, generates daily CHO that contributes to the daily allowed CHO in a KD regimen [211]. The CHO elevation in such cases might go unnoticed and might require dietary plans with lower CHO content. It is indeed challenging for a health care practitioner to be qualified enough to spot these cases and adjust their suitable diet accordingly. Detailed assessment before initiating a KD is mandatory [212] and should include the entire medical history, nutritional status, laboratory tests, and any contraindications to start the KD [212].

The suitability of the KD implementation varies between individuals. Detailed medical guidance is required for some patients, such as people with diabetes and those at high risk of developing complications, like hyperlipidemia, severe liver and renal diseases, and metabolic disturbances, to minimize unnecessary side effects and improve their outcomes [212,213]. Despite the detailed reporting of the contraindications of the KD in the literature [154], studies regarding these concerns among vulnerable individuals are lacking. Nowadays, the KD is commonly initiated by experienced nutritionists; however, ascertaining whether all of them possess the sufficient qualification to distinguish the suitable candidates for a KD from those at risk is a challenge on its own.

A few other challenges were reported by a study that evaluated the worldwide use of the KD. In countries with CHOs, especially rice, as a principal constituent of their diet, following a KD was complicated [214]. Sometimes, a clear nutritional information label is not provided on ready-made food items and ensuring their components might be difficult [214]. In addition, financial issues in some regions might stand in the way of following, and adhering to, a KD [214]. A randomized cross-over study compared the adherence of individuals on the KD to Mediterranean diet during periods where the diet was readily provided and when self-prepared by participants and found a lower dietary adherence when participants were responsible for purchasing their own meals [215]. After all, the KD is a therapeutic intervention and can face various obstacles during its application. The ongoing research might be promising to overcome a few of the challenges mentioned above.

## 5. Discussion

**Figure 2:**
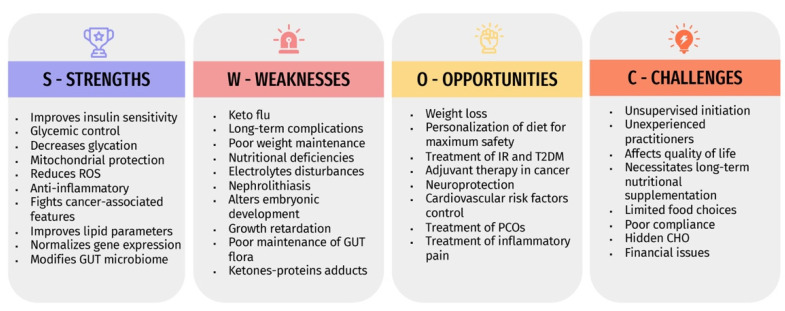
SWOC analysis of the ketogenic diet; the strengths, weaknesses, opportunities, and challenges are presented in the form of concise points. ROS; reactive oxygen species; GUT, digestive tract; IR, insulin resistance; T2DM, type 2 diabetes mellitus; PCO, polycystic ovarian syndrome; CHO, carbohydrates.

The expanding global prevalence of various diseases warrants revealing the safety and efficacy of any therapeutic approach, including dietary interventions. The KD is becoming promoted nowadays as an effective and satisfying weight reduction method by the public. However, the initiation of unsupervised KD protocol is common and might result in more harm than benefit. Due to the insufficient comprehensive analysis of all aspects of the KD, we performed a SWOC analysis that aimed to clarify the potential advantages of KD, drawbacks, value in certain disorders, and its associated challenges, and how to deal with them. Figure 2 summarizes the SWOC analysis results.

The discussed mechanisms of action of the KD at the cellular, molecular, biochemical, and immunological levels opened the door to its employment in a protective and therapeutic modality. Inducing nutritional ketosis by a well-formulated KD can provide huge benefits for patients with obesity, IR, T2DM, and MetS, which not only improves glycemic control and overall health, but ultimately impacts cardiovascular health and helps in treating other endocrinological disorders such as PCOS. However, the long-term safety of the KD on CV health cannot be ascertained, which should be kept in mind if the KD was carried out in those with high CV risk.

Chronic inflammation and oxidative stress are two key elements in the development of various disorders [216]. Nutritional ketosis and KBs were proven to successfully combat mitochondrial dysfunction, reduce ROS formation, activate antioxidant pathways, and suppress inflammation. These mechanisms have given optimism for those with disorders of exaggerated inflammatory processes, such as neurodegeneration and cardiometabolic disorders [217], and in cases of mitochondrial malfunction. KD was also shown to be protective of the mitochondrial genome from oxidative damage [54]. Furthermore, the ability of KD to build an overall unfavorable metabolic environment for cancer cells was able to aid in shortcutting the tumors’ nourishment, as malignant cells fail to thrive on ketones [53]. This has given faith in enhancing chemotherapeutic effects and positively influencing those with glioblastoma, gastric cancers, and other types of malignancies.

The significance of the KD in improving neurological disorders resides in its antioxidant, anti-inflammatory, and mitochondrial restoration properties. The established role of KD as a non-pharmacological agent for treating multiple forms of epilepsy [32,168] have given it various opportunities to be implemented in other neurological disorders, such as Alzheimer’s [218], Parkinson’s [113], and multiple sclerosis (MS).

The significant advantages offered by the KD poses the question: is the KD very innocent to provide weight loss values and options for treating various conditions without complications? The numerous KD-related drawbacks and challenges explained clarify that the KD is not for everyone and should be initiated with caution. Short-term adverse effects such as keto flu, hair loss, palpitations, or leg cramps may be tolerable by most people [108]; however, long-term complications such as cardiomyopathy, renal stones, and prolonged nutritional deficiencies may have detrimental effects [111,112,113]. Furthermore, many ketosis-related complications might be associated with metabolic acidosis, which might be dangerous in some cases, such as pregnancy and breastfeeding [139]. Additionally, the blood pressure and blood glucose-lowering effects among hypertensives and diabetics might induce attacks of life-threatening hypotension or hypoglycemia if not properly monitored [110]. Moreover, a meta-analysis of observational studies that assessed the effects of a long-term KD has revealed its association with a higher risk of all-cause mortality [219]. Aside from its drawbacks, the KD faces multiple challenges that may interfere with its outcomes, such as changing quality of life, maintaining sufficient nutrients, proper monitoring of ketosis, and ensuring full awareness of its adverse effects by health care providers [163,204,205,212].

In summary, the KD shows variable physiological and metabolic responses between individuals that are dependent on various factors, such as diet compliance, co-existing medical illnesses, financial issues, and the level of professionalism of health care practitioners initiating the KD. Whether KD benefits for an individual outweigh potential risks should be well-founded before deciding to follow a KD.

According to our knowledge, no comprehensive work was dedicated before to improving KD implementation under the umbrella of a SWOC analysis. Our review was the first to provide an opportunity for healthcare practitioners and nutritionists to design a personalized approach before initiating a KD that is well-formulated to reveal the full opportunities of a KD to take advantage of, and to highlight possible future challenges that one can face when implementing a KD. Furthermore, it prevents disregarding the pitfalls of a KD that could be more harmful than beneficial and encourages healthcare providers to avoid being short-sighted when making healthcare-related decisions. Moreover, understanding the detailed strengths and weaknesses of all aspects of the KD ensures reserving these types of dietary regimens for those who would actually benefit from the opportunities it provides, with maximum safety measures.

## 6. Conclusions

The SWOC analysis highlighted the main strengths and weaknesses of KD. It summarized all points of interest that help physicians decide the suitability of KD for specific individuals and its inappropriateness to others. Conclusively, in a novel context, this SWOC analysis provides the possibility to analyze each individual’s situation, catch opportunities that would mostly benefit them, execute all possible challenges they might encounter, and accordingly, plan an individual-specific therapeutic approach.

## Data Availability

Not applicable.
